# Genome Sequence of the Bacterium *Bifidobacterium longum* Strain CMCC P0001, a Probiotic Strain Used for Treating Gastrointestinal Disease

**DOI:** 10.1128/genomeA.00716-13

**Published:** 2013-09-12

**Authors:** Hongjing Yu, Lu Liu, Zhen Chang, Shasha Wang, Bin Wen, Peijun Yin, Datao Liu, Bei Chen, Jundong Zhang

**Affiliations:** Research and Development Center of Shanghai Sine Pharmaceutical Co., Ltd., Shanghai, China

## Abstract

*Bifidobacterium longum* subsp. *longum* CMCC P0001, a standard probiotic strain in China, has been widely used in clinical medicine for more than 20 years. Here we report the genome features of *B. longum* strain CMCC P0001.

## GENOME ANNOUNCEMENT

*Bifidobacterium longum* subsp. *longum* strain CMCC P0001, isolated from feces of healthy children, has been commercially used in the probiotic compound BIFICO (Shanghai Sine Pharmaceutical Co., Ltd., Shanghai, China) for more than 20 years (1, 2). In 2011, the strain was designated a standard strain for probiotic production by the China Medical Culture Collection Center (CMCC) with the assigned number CMCC P0001.

Probiotics and their metabolites have been demonstrated to be crucial to human health. Among all probiotics, those of the genus *Bifidobacterium* are indeed remarkable, because they play important roles in preventing infection, enhancing immunity, inhibiting the growth of pathogenic bacteria, and treating inflammatory diseases (3). Here, the draft genome sequence of the standard probiotic strain *B. longum* CMCC P0001 is presented.

The genome sequence of CMCC P0001 was determined by the use of the Illumina HiSeq 2000 system with paired-end and shotgun libraries (176× coverage). As a result, a total of 5,722,028 reads with an average length of 74 bp were assembled into 136 contigs using SOAP denovo-1.04 (4, 5). Protein-coding genes were predicted by GLIMMER 3 (6). Protein functions were annotated by use of a sequence similarity search using BLAST programs (7) against the proteins of the other *B. longum* strains and the nonredundant protein database of the NCBI. tRNAs and rRNAs were identified by tRNAScan-SE (8) and BLAST (9), respectively.

The draft genome sequence of *B. longum* CMCC P0001 consists of a 2,418,214-bp circular molecule without any plasmids. The overall G+C content of the genome is 59.75%. CMCC P0001 harbors 54 tRNA genes. A total of 1,569 coding sequences (CDS) were predicted in the genome and 1,391 (88.7%) CDS were predicted to be functional, whereas protein functions for 178 (11.3%) of the CDS were classified to be unclear.

A gene encoding a serpin (serine protease inhibitor) with almost 100% similarity to that of NCC2705 (10) was found in the genome sequence. As a potential probiotic effector molecule, the serpin may contribute to the immunomodulation of this *B. longum* strain. Furthermore, a large number of predicted proteins (>8% of the total predicted proteins) encoded in the genome were in the carbohydrate transport metabolism category. The ability to enhance carbohydrate transport metabolism likely contributes to the competitiveness and persistence of bifidobacteria in the colon (11).

The availability of the whole-genome sequence of CMCC P0001 will facilitate further analysis and understanding of the health-promoting characteristics of the probiotic strain *B. longum* CMCC P0001.

### Nucleotide sequence accession number.

The draft genome sequence of *B. longum* CMCC P0001 has been deposited at GenBank under the accession number APVE00000000.
